# Dialysed Iron

**Published:** 1877-07

**Authors:** 


### Dialysed Iron.

We would call the attention of physicians to this new pharmaceutical preparation. It has been long known to the experimental chemists, but until lately has not been manufactured in sufficient quantity to make it a commercial product. By the method employed by Wyeth & Bro. a dark brown liquid, having little styptic taste is made. This in connection with the fact that it does not blacken the teeth, nor disturb the alimentary canal, will recommend it above ordinary preparations of iron. It cannot, however, be given in composition with any other drug, as it is liable to precipitation, the addition of a few drops of undistilled water clouding the solution. In all cases where the use of iron is indicated this will be found to be the most agreeable mode of prescription. It is a pure hydrate and as efficient as the sesquioxide in all cases of poisoning, when the latter is useful, besides having the additional advantage of being always ready for use.

